# Preparing for Future Group B *Streptococcus* Vaccines: Perspectives of Pregnant Women in Australia

**DOI:** 10.3390/vaccines14090807

**Published:** 2026-09-14

**Authors:** Prabha H. Andraweera, Emma Jeffs, Bing Wang, Helen S. Marshall

**Affiliations:** 1 School of Medicine, College of Health, Adelaide University, Adelaide, SA 5000, Australia; bing.wang@adelaide.edu.au (B.W.); helen.marshall@adelaide.edu.au (H.S.M.); 2 Vaccinology and Immunology Research Trials Unit, Women’s and Children’s Health Network, Adelaide, SA 5006, Australia; 3 Clinical Practice Development Unit, Women’s and Children’s Health Network, Adelaide, SA 5006, Australia; emma.jeffs@sa.gov.au

**Keywords:** Group B *Streptococcus* (GBS), vaccine, immunisation, acceptance, pregnancy

## Abstract

Background: Vaccines for Group B *Streptococcus* (GBS) intended for pregnant women are currently in advanced stages of clinical trials. Conducting preparatory research now can help identify factors influencing GBS vaccine acceptance and potential challenges to future implementation. Methods: This qualitative study recruited participants nationally through social media and antenatal clinics of a maternity hospital in South Australia between December 2024 and August 2025. Eligible participants were pregnant women aged ≥18 years residing in Australia. Online interviews and focus group discussions (FGDs) were held to explore pregnant women’s knowledge and perceptions of GBS, experiences with current screening and management strategies, and views on future maternal GBS vaccination. Twenty-five women participated in FGDs and six in individual interviews; FGD group sizes ranged from two to five participants. Data were analysed using inductive content analysis. Results: A total of 31 pregnant women participated, aged 19–43 years. Most participants (51.6%) were from South Australia, 87.1% had tertiary education, and 51.6% were employed in the health sector. Five themes were identified: (1) infant wellbeing is the dominant driver of vaccine acceptance, (2) maternal vaccination is preferred over current antibiotic prophylaxis, (3) perceived uncertainty about vaccine safety and knowledge gaps fuel caution, (4) accessible and flexible vaccination delivery models are desired, and (5) multimodal, woman-centred communication delivered through trusted healthcare providers is required. Conclusions: Findings highlight the importance of addressing knowledge and safety concerns and incorporating accessible, flexible and woman-centred approaches into the planning of future maternal GBS vaccination programmes.

## 1. Introduction

Group B *Streptococcus* (GBS) is a leading cause of neonatal morbidity and mortality worldwide. In 2020, it was estimated that approximately 19.7 million pregnant women were colonised with GBS [1]. Maternal colonisation can lead to maternal GBS infection, preterm delivery, stillbirth and vertical transmission of GBS to the infant during labour or delivery, resulting in early-onset (0–6 days) GBS disease in the newborn baby. Late-onset GBS (7–89 days) in infants which occurs after the first week is less likely to be due to vertical transmission. Invasive GBS disease in infants can present as sepsis, pneumonia or meningitis and can have long-term sequelae including neurodevelopmental impairment [1].

Current GBS preventive strategies in high-income countries including Australia focus primarily on antenatal screening and intrapartum antibiotic prophylaxis for women who test positive. The Royal Australian and New Zealand College of Obstetricians and Gynaecologists guidelines recommend all maternity services should have an established plan for prevention of early-onset GBS infection. Universal culture-based screening, using combined low vaginal and/or anorectal swab at 35–37 weeks of gestation or 3–5 weeks prior to anticipated delivery in high-risk pregnancy such as poorly controlled diabetes and multiple pregnancy, or a clinical-risk-factor-based approach are both acceptable strategies for reducing early-onset GBS disease [2]. Self-collection of vaginal and rectal specimens is supported as an option for GBS screening in Australia; however, it varies across states, territories and individual maternity services. While this screening strategy has reduced the incidence of early-onset GBS disease, it does not prevent all cases and has no impact on late-onset disease. In addition, intrapartum antibiotic usage raises concerns regarding antimicrobial resistance and disruption of the infant microbiome [3]. For pregnant women, antibiotic prophylaxis may also influence labour experiences including need for intravenous access, timing of hospital admission for delivery and postnatal monitoring of infants.

Maternal immunisation is an effective public health strategy for preventing infectious diseases in early infancy by inducing maternal antibodies that are transferred via the placenta to the foetus [4]. Vaccination in pregnancy is now well established for influenza, pertussis and respiratory syncytial virus (RSV). Within this context, the World Health Organisation (WHO) has developed a maternal immunisation framework to guide vaccines intended for use in pregnancy. This framework emphasises that successful maternal immunisation programmes require not only safe and effective vaccines but also health system readiness, integration into antenatal care and acceptability among pregnant women [5]. The WHO has identified GBS as a priority pathogen for maternal vaccine development and has developed preferred product characteristics to guide development of maternal GBS vaccines [6]. There are no maternal GBS vaccines currently licenced or recommended for use. However, several candidate vaccines are in advanced stages of clinical trials.

The most advanced candidate is an investigational hexavalent capsular polysaccharide CRM197 conjugate vaccine (GBS6) developed by Pfizer. GBS6 has demonstrated acceptable safety and immunogenicity in Phase 2 studies in pregnant women, including women living with HIV, with vaccine-induced antibodies transferred to their infants [7,8]. The vaccine is currently being evaluated in a global Phase 3 trial assessing safety, tolerability, and immunogenicity (BEATRIX, NCT07160244), which began recruitment in August 2025 across sites in North America, Europe, Africa and Asia, with primary completion expected November 2028. A recombinant protein-based candidate developed by MinervaX, GBS-AlpN, also referred to as GBS-NN/NN2, has completed Phase 2 trials (NCT04596878) in pregnant women. GBS-AlpN had an acceptable safety profile and was immunogenic when administered to pregnant women, supporting its progression to Phase 3 trials [9]. As maternal GBS vaccines move closer to potential licensure and inclusion in antenatal care programmes, understanding pregnant women’s knowledge, perceptions, concerns, and acceptance of GBS vaccination will be essential to support successful uptake and implementation strategies.

The aim of this study was therefore to explore pregnant women’s knowledge and perceptions of GBS, experiences with current screening and management strategies, and views on future maternal GBS vaccination in Australia.

## 2. Materials and Methods

### 2.1. Study Design and Setting

This was a descriptive qualitative study involving semi-structured interviews and focus group discussions (FGDs) to explore pregnant women’s views on future maternal GBS vaccines. Descriptive qualitative research develops findings that remain close to the participant experience as they describe it, and is a useful methodology for generating clear, tangible recommendations [10]. Interviews and FGDs were held online with women residing in Australia.

### 2.2. Sampling and Recruitment

Participants were recruited through the Facebook account of the authors’ university (Adelaide University), at antenatal waiting rooms at the Women’s and Children’s Hospital in Adelaide, Australia, and through snowball sampling [11,12]. The Facebook advertisement provided a link directing women to the participant information sheet and consent form on the REDCap version 17 electronic data capture tool hosted on the Adelaide University server. A research nurse discussed the study with pregnant women at antenatal waiting rooms and provided the link to the participant information sheet and consent form on RedCap. Women were eligible if they were aged ≥18 years, residing in Australia and were pregnant at the time. None of the members of the research team had a role in the clinical care or management of the participating women. Participants were recruited between December 2024 and August 2025.

### 2.3. Data Collection

Demographic information, parity and gestational age at registration were collected on RedCap. Interviews and FGDs took place via the Adelaide University online platform Zoom Video Communications (San Jose, CA, USA) and were facilitated by an experienced qualitative researcher PA (female, Doctor of Philosophy, researcher) or EJ (female, Doctor of Philosophy, nurse). Online interviews and FGDs allowed accessibility to the study for women across Australia. Interviews and FGDs were conducted at a time convenient to participants. A semi-structured interview guide (File S1) was used to facilitate the interviews and FGDs. The first component of the interview/FGD explored the level of knowledge about GBS in pregnancy and consequences for the newborn baby. The second component explored the level of understanding of the current GBS screening methods, views on antibiotic prophylaxis during labour, antibiotics for the newborn baby and information needs. The third component explored preferences and acceptability towards future maternal GBS vaccines. The interviews were conducted in English and audio recordings were anonymised and transcribed.

### 2.4. Ethical Considerations

Ethics approval was provided by the Women’s and Children’s Hospital Human Research Ethics Committee (2024/HRE00205, 21 September 2024). Participant consent was obtained before the interview and consent could be withdrawn at any point. Consent was also obtained for recording of the interviews/FGDs. The video recordings were deleted after transcription. Participants received a $50 Coles Group and Myer voucher for their time. Researchers facilitating FGDs recognised that speaking about pregnancy could potentially cause distress to participants. Researchers maintained a commitment to fostering a safe interview environment where participants could decline answering questions or take a break when needed.

### 2.5. Data Analysis

Reflective notes were taken by the facilitators during and following the FGDs and interviews and were discussed in regular debriefing meetings held throughout the data collection period. These notes were used to inform the researchers’ understanding of the context of the discussions and were considered alongside the transcripts during analysis. Anonymised transcripts were imported into the software programme NVivo (version 15) and analysed using inductive content analysis [13]. Two researchers (PA and EJ) independently coded the transcripts, with the lead facilitator (PA) undertaking initial line-by-line coding of the early transcripts. Coding was subsequently discussed between the two coders, and the coding framework was reviewed and refined through regular meetings. Minor additions or changes to the coding framework were discussed with the broader research team (BW, HM) and, where appropriate, applied retrospectively to previously coded transcripts. Findings were developed by grouping related codes into themes, which were reviewed, refined and discussed during research team meetings. Transcripts or preliminary findings were not returned to participants for comment or verification. Given the group-based nature of the focus group discussions and the potential for disclosure of other participants’ comments, participant transcript verification was not undertaken. Instead, credibility was enhanced through the use of two coders, regular analytic discussions, reflexive notes, and review of the coding framework and emerging themes by the broader research team. The sample size was determined based on the number of participants required to obtain a range of perspectives relevant to the research question and the emergence of data saturation. Data collection continued until the research team determined that no substantively new codes or themes were emerging from subsequent interviews or focus group discussions [14].

## 3. Results

A total of 43 women consented to participate in the study, of whom 31 were successfully recruited (Table 1). A total of 25 participants took part in FGDs, with group sizes ranging from two to five participants. Two FGDs comprised two participants each. In addition, six participants took part in individual interviews. The remaining women who consented (*n* = 12) were unable to participate due to difficulties identifying convenient times for discussions (*n* = 6), early delivery prior to participation (*n* = 2), medical or pregnancy-related complications requiring hospital admission (*n* = 1) or non-attendance at scheduled sessions (*n* = 3). All participants spoke English as their first language. There was representation from all six Australian states and one territory: South Australia (*n* = 16), New South Wales (*n* = 5), Victoria (*n* = 3), Western Australia (*n* = 3), Queensland (*n* = 2), Tasmania (*n* = 1), and the Australian Capital Territory (*n* = 1). The participants were aged between 19 and 43 years, and 12 were experiencing their first pregnancy. Two participants were in the first trimester of pregnancy, 13 were in the second trimester, and 16 were in the third trimester. Each interview lasted between 30 and 40 min and each FGD lasted between 50 and 60 min.

### 3.1. Themes

**Figure 1:**
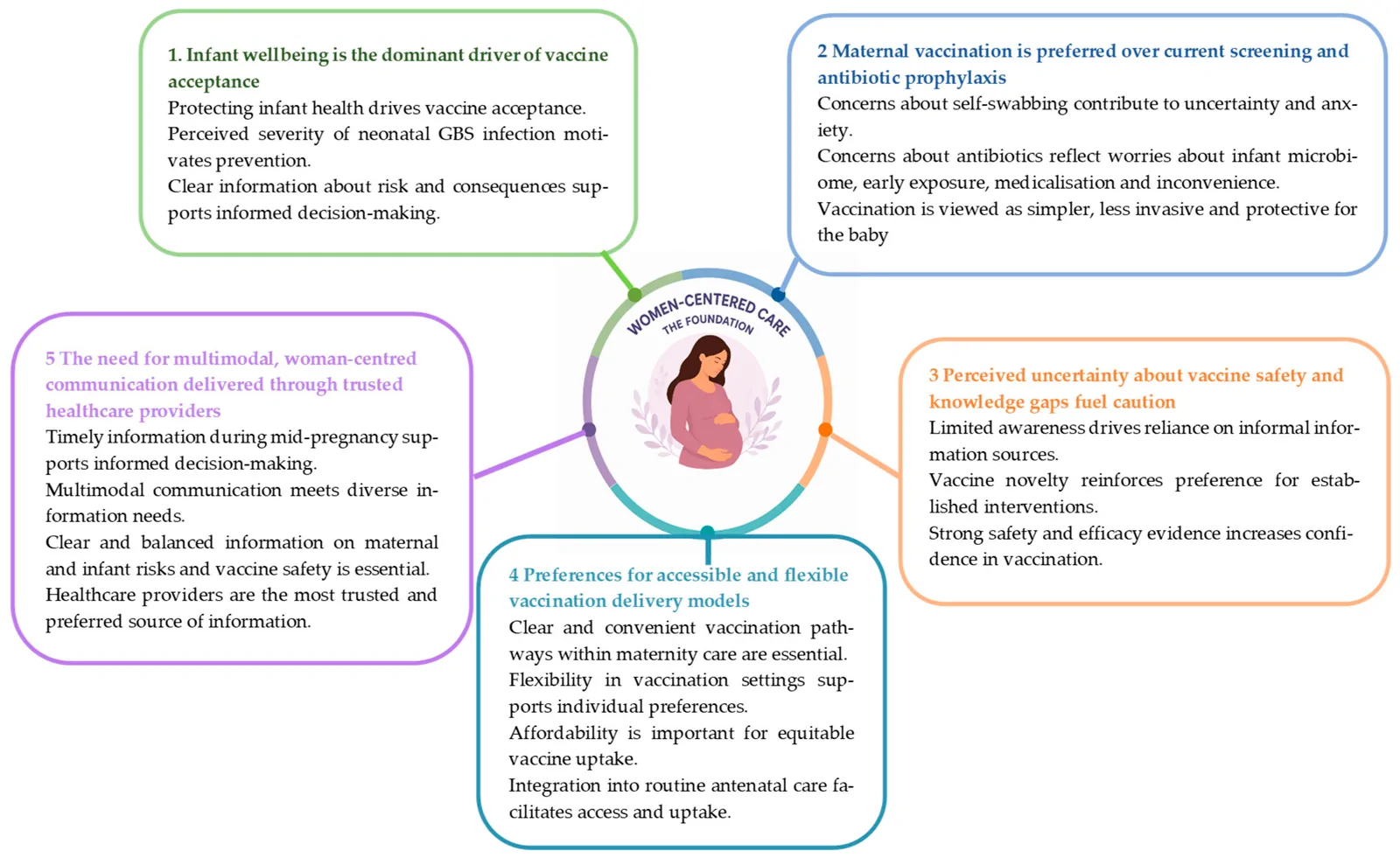
Thematic framework of factors influencing pregnant women’s perspectives on a future Group B Streptococcus (GBS) vaccine.

We identified five primary themes, summarised in Figure 1 and described in detail below.

#### 3.1.1. Infant Wellbeing Is the Dominant Driver of Vaccine Acceptance

Participants’ willingness to consider a future GBS vaccine in pregnancy was strongly driven by perceptions of baby wellbeing and safety. Women consistently framed vaccine acceptability in terms of protecting their infant, often prioritising infant outcomes over their own health. As one participant explained, “I obviously want to protect my baby as much as I can” [P6], while another stated they would “want to know more about what it did for the baby… more so than myself” [P1], highlighting an infant-centred decision-making approach.

Perceived severity of neonatal GBS infection was a key factor supporting vaccine uptake. A few participants (*n* = 4) recognised the potential seriousness of GBS infection in newborns, describing it as potentially life-threatening and therefore an important condition to prevent. As one participant explained, “My broad understanding is that it can be life threatening to the baby” [P3]. Similarly, concerns about adverse infant outcomes guided acceptance of preventive strategies, with one participant reflecting, “I thought, if something happened to my baby and I hadn’t taken steps to prevent it, I’d be devastated” [P9]. This strong desire to avoid harm to the baby extended to vaccine considerations, where prevention was viewed as a means of avoiding distressing neonatal complications.

Participants also identified a need for clearer information on the burden and likelihood of neonatal infection to support informed vaccine decision-making, including uncertainty around “how likely it is to pass on to baby” [P6] and “how serious is the GBS infection for newborn babies” [P5]. Concerns about downstream consequences of neonatal infection and treatment were also raised, including the need for intravenous antibiotics, breastfeeding disruption, infant distress, and potential medication side effects. Despite recognising that infection may not always result in harm, participants emphasised the unpredictability of outcomes, noting that GBS “can cause no harm at all… but it can be quite harmful if it’s passed on to bubs” [P2].

Overall, anticipated acceptance of a future maternal GBS vaccine was closely linked to the perception that it would safeguard infant health, with decision-making influenced by minimising potential harm to infant.

#### 3.1.2. Maternal Vaccination Is Preferred over Current Screening and Antibiotic Prophylaxis

Participants frequently compared a future maternal GBS vaccine with current prevention strategies, including antenatal screening and intrapartum antibiotic prophylaxis. Concerns regarding self-collected swabs, limited information provision, and the perceived negative impacts of antibiotics contributed to a preference for vaccination among many women.

Several participants (*n* = 7) expressed uncertainty about correctly performing self-collected GBS swabs. One participant stated, “I don’t have any problems with doing a swab myself… but that leaves a window… people not doing it correctly” [P1], while another commented that it would be “fine with a practitioner doing it just to make sure that it’s done right” [P6]. Women also described the process as awkward and anxiety-provoking, particularly during pregnancy. As one participant explained, “It’s a bit awkward to do on yourself because you can’t really see what you’re doing” [P8]. Others highlighted limited education surrounding screening, with one participant stating, “there was no fact sheet, there was nothing” [P21].

Participants additionally raised concerns regarding intrapartum antibiotics, particularly potential impacts on the infant microbiome, early antibiotic exposure, and the medicalisation of labour. One woman reflected, “I was thinking about the microbiome benefits of a vaginal birth. If you have antibiotics, you might reduce that benefit” [P9]. Participants also described the inconvenience of intravenous antibiotics during labour, with one woman with GBS in a previous pregnancy commenting, “Labouring with the cannula in was really frustrating” [P2].

These concerns contributed to a preference for vaccination over screening and antibiotics among the majority of participants. Vaccination was commonly perceived as a simpler and less invasive preventative approach. One participant explained, “With antibiotics… it’s an annihilator, whereas a vaccine is more of a protector” [P1]. Another stated, “I’d probably prefer the vaccine over the antibiotics… because antibodies would go to bubs” [P2], while a third described vaccination as “more straightforward than having the swab and then getting the result, and then having the antibiotics” [P3]. Three women preferred antibiotics over a vaccine, with one stating, “I’d prefer antibiotics, something I know, rather than a new vaccine I’m unsure about” [P10]. Overall, the majority of participants preferred maternal vaccination as a simpler and more acceptable approach to GBS prevention than current screening and intrapartum antibiotic prophylaxis.

#### 3.1.3. Perceived Uncertainty About Vaccine Safety and Knowledge Gaps Fuel Caution

Participants generally reported limited prior awareness of GBS in pregnancy, describing it as information they had only recently encountered during their current or previous pregnancies. Several women indicated they “actually didn’t know it was a thing before I was pregnant” [P12], while others noted that their understanding had developed only through pregnancy-related information-seeking, stating, “I just know from what I’ve read this pregnancy and last pregnancy” [P4]. Informal sources such as online pregnancy forums and social media were commonly referenced, with one participant noting, “I saw on a pregnancy Facebook group, someone who just tested positive to it, and was asking questions about it” [P6]. Many women described actively searching for information to compensate for this lack of baseline knowledge, including “I’ve done a little bit more sort of Googling… just to understand a little bit more about it” [P2] and “I started reading about the statistics… the actual risk of the baby becoming symptomatic” [P1]. Despite this information-seeking, some participants still reported uncertainty about neonatal consequences, with one stating, “I actually don’t know anything about that [consequences for infant]” [P8].

This limited baseline knowledge led to uncertainty about a future vaccine, where safety and evidence of effectiveness were central to acceptability. Participants frequently emphasised the need for robust clinical data before acceptance, with one noting, “I’d want to know how it works, especially if most people already carry Group B Strep. What does the vaccine do if you’ve already been exposed? I’d want to know it’s safe and effective” [P9], while others highlighted reliance on evidence, stating, “I think science and statistics and studies speak volumes” [P1]. Trust in regulatory approval was considered important but not sufficient on its own, with participants still seeking details on study design and population exposure, including “what population group for how long? How big a study?” [P5] and “how many people participated, and the safety data” [P10]. Concerns were also expressed regarding the novelty of a potential vaccine, with some indicating a preference for established interventions such as antibiotics or long-standing vaccines, describing a willingness to “prefer to stick to the tried and tested antibiotics until I saw a lengthy period of proof that the vaccine is effective and doesn’t have any side effects” [P22] and a tendency to “wait until… everyone else had tried it first” [P21].

Despite these concerns, some participants indicated conditional acceptance of a future vaccine if sufficient safety and efficacy data were available, with one stating, “If it’s been deemed safe for pregnant women, then that’s pretty well all I’d need to know” [P3]. Others expressed their trust in established vaccines with hesitancy toward newer ones, noting, “I’ve had plenty of vaccines in my life… I do trust them. But I don’t trust ones that have just come onto the market” [P22]. Overall, hesitancy toward a future GBS vaccine was influenced by limited baseline awareness of GBS in pregnancy combined with strong demands for evidence of safety, efficacy, and long-term clinical experience.

#### 3.1.4. Preferences for Accessible and Flexible Vaccination Delivery Models

Participants emphasised that the accessibility and delivery setting of a future GBS vaccine would be important factors influencing uptake in pregnancy. Preferences varied regarding appropriate service delivery models, with some women indicating a hospital-based setting as the most suitable option, stating, “I think it needs to be given only in a hospital setting” [P1]. Others, however, highlighted the importance of clarity and flexibility around where vaccination could be accessed, questioning whether it would be available through general practice or pharmacies, or whether it would require attendance at a hospital-based service: “And is it something we could get at the GP. Or is it something we could get at the chemist? Or is it something special that we’d have to go to hospital for” [P13]. This uncertainty reflected a broader need for clear pathways to access maternal vaccination services.

Cost was also identified as a potential barrier to uptake, with participants suggesting that financial burden could discourage some women from receiving a vaccine in pregnancy. One participant noted, “If there’s going to be a big cost with a vaccination could be a bit of a hindrance for some people” [P12]. Overall, participants indicated that uptake of a future GBS vaccine would be facilitated by clear, convenient, and affordable access pathways integrated into existing maternity care services.

#### 3.1.5. The Need for Multimodal, Woman-Centred Communication Delivered Through Trusted Healthcare Providers

Participants highlighted that communication about GBS and a future vaccine should be delivered through clear, timely, and multimodal strategies embedded within routine antenatal care. Many women suggested that information should be introduced at specific points in pregnancy when they are most receptive, commonly around the mid-pregnancy period. Suggested timing included “around the late 20-week mark” [P2] and “like the 24 week mark” [P21], with others indicating preference for early antenatal visits such as the first or second appointment, where “I would like to be told about it” [P6]. However, some participants also noted that early appointments can be overwhelming due to the volume of information provided, suggesting that written materials could complement verbal discussions, for example, “At the first appointment, I feel like there’s so much stuff that gets thrown at us… maybe a brochure could be given at that visit” [P7].

Written resources were consistently valued as an important addition to verbal communication, allowing women time to revisit information after appointments. Participants described preferences for concise, accessible materials such as “a fact sheet… A4 page would probably be best for me” [P21], and highlighted the importance of clarity and inclusivity, including “Easy to read and easy to understand… different languages as well” [P23]. Brochures were seen as particularly useful when integrated with existing antenatal resources, such as “a pamphlet with your orange book” [P3], and were considered helpful in prompting further questions during clinic visits. Women also expressed interest in trusted digital and community-based sources, including reputable pregnancy websites and podcasts, with one participant noting “Raising Children or Great Birth Rebellion… are trusted spaces for many women” [P9].

The importance of content was also emphasised, with participants seeking clear explanations of both maternal and infant risk, “What’s the risk to me? What’s the risk to my child?” [P21], as well as detailed information on vaccine safety, mechanism, and study follow-up. Some also highlighted the need to address common misconceptions, including concerns about vaccine components and misinformation, with suggestions that explaining “whether it’s a live or inactive vaccine” [P10] and proactively addressing myths could improve understanding and reduce hesitancy. Participants further noted that clarity around current management strategies and their implications, such as antibiotic use, microbiome effects, and mode of birth considerations, would support informed decision-making.

Healthcare providers were identified as the most trusted source of information, with women expressing strong preference for receiving information from midwives, GPs, or obstetricians rather than mass media or social platforms. As one participant stated, “I rely more on my obstetrician or midwife to tell me if I need to do anything” [P8], while others emphasised that advice from trusted clinicians would be sufficient to guide uptake if safety was assured. Broader public campaigns were viewed cautiously, with concerns that they could introduce misinformation or create opportunities for negative opinions, whereas clinician-led communication was seen as more credible and reassuring.

Overall, participants favoured a woman-centred, multimodal communication approach combining timely verbal discussions, written materials, and trusted digital resources delivered through antenatal care pathways. This approach was perceived as essential to support informed decision-making and facilitate acceptance of a future maternal GBS vaccine.

## 4. Discussion

This qualitative study explored pregnant women’s perceptions of GBS in pregnancy and views on future maternal GBS vaccination. Five themes highlighted that infant wellbeing was central to vaccine decision-making, while limited awareness and concerns about the safety and effectiveness of a novel vaccine could contribute to caution. Participants also identified practical considerations for future implementation, including convenient access to vaccination and clear, woman-centred communication delivered through trusted healthcare providers.

A key finding was that participants viewed protection of the infant as an important consideration when deciding whether to receive a future GBS vaccine. This is consistent with previous maternal immunisation research demonstrating that perceived protection of the foetus or newborn is an important driver of vaccine acceptance [15,16]. Participants also recognised the potential severity of neonatal GBS infection but wanted clearer information about the likelihood and consequences of infection. This suggests that future communication should clearly explain both the burden of neonatal GBS disease and the potential benefits and limitations of maternal vaccination.

Limited awareness of GBS was also prominent. Many participants had encountered information about GBS only during pregnancy or through informal sources, and several reported uncertainty about neonatal consequences and current prevention strategies. These findings are consistent with Sosa et al. [17] who reported low awareness of GBS among pregnant women and found that providing information about GBS increased willingness to accept a hypothetical vaccine. Our findings extend this work by illustrating how limited baseline knowledge and uncertainty may shape the questions women want answered before considering a future vaccine, particularly regarding disease severity, vaccine safety and effectiveness. Together, these findings support the importance of providing evidence-based information before and during pregnancy, rather than assuming that women will be familiar with GBS or its prevention.

Concerns about the safety and evidence supporting a novel vaccine were an important consideration. Participants wanted information about clinical trial evidence, safety, effectiveness and follow-up before making decisions about vaccination. Similar concerns about the safety of a new GBS vaccine have been reported previously [17], reinforcing the importance of transparent communication about the evidence supporting vaccine use in pregnancy. Providing information in layers, with concise information for women who prefer brief explanations and access to more detailed evidence for those seeking further information, may help accommodate different information needs.

Trust in healthcare providers was an important enabling factor. Participants identified midwives, GPs and obstetricians as preferred sources of information and indicated that a recommendation from a trusted healthcare professional could influence their decision. This is consistent with evidence that healthcare provider recommendation is a strong predictor of maternal vaccine uptake [18]. However, participants also described a need for consistent and accessible information, suggesting that implementation should include education and resources for healthcare professionals so that they are confident discussing GBS vaccination and can provide consistent advice across antenatal care settings.

The findings have several implications for planning a future maternal GBS vaccination programme in Australia. First, vaccination should be integrated into existing antenatal immunisation pathways where feasible, alongside established maternal vaccines such as influenza, pertussis and RSV, to provide convenient opportunities for vaccination. Participants expressed preferences for different delivery settings, including hospitals, general practices and pharmacies; therefore, clear and consistent information about where vaccination can be obtained will be important. Flexible delivery models may also reduce practical barriers for women receiving antenatal care across different settings.

Second, affordability should be considered as part of implementation planning. Participants identified vaccine cost as a potential barrier. In Australia, affordability would depend in part on decisions regarding public funding and inclusion in the National Immunisation Programme (NIP). For a new vaccine to be supplied through the NIP, it must undergo regulatory assessment by the Therapeutic Goods Administration, receive a Pharmaceutical Benefits Advisory Committee (PBAC) recommendation, undergo pricing and government approval processes, and subsequently be listed and procured through the NIP. The Australian Technical Advisory Group on Immunisation (ATAGI) provides clinical and technical advice to inform these processes, including advice accompanying vaccine submissions to PBAC. Consideration of the clinical effectiveness, safety, cost-effectiveness and budgetary implications of a future GBS vaccine will therefore be important for determining whether vaccination can be offered equitably and without cost acting as a barrier.

Third, implementation should incorporate a coordinated communication strategy. Rather than relying on a single source or encounter, information could be provided through routine antenatal consultations and supported by concise written and digital resources that women can revisit in their own time. Healthcare professionals should be equipped with consistent, evidence-based resources addressing disease burden, vaccine safety and effectiveness, and common questions. Communication should be timely, accessible and woman-centred, while allowing women access to more detailed information where desired. These approaches are particularly relevant given that current Australian maternal vaccination programmes already provide established opportunities for integrating information and vaccination into antenatal care.

This study has several strengths. The use of both focus groups and individual interviews enabled exploration of shared and individual perspectives. Recruitment across multiple Australian states increased variability in participant experiences, supporting transferability. Reflexive discussions throughout data collection and analysis, independent coding by two researchers, and review of emerging themes by the broader research team strengthened the analytic process.

Several limitations should also be acknowledged. Although participants were recruited from multiple Australian states, the sample was weighted towards South Australia, and included a substantial proportion of women aged over 30 years, women with higher educational attainment and employment in the healthcare sector. These characteristics may have contributed to greater baseline health knowledge and familiarity with healthcare systems and may limit the transferability of findings to the broader pregnant population. Recruitment through voluntary participation and the Adelaide University Facebook page may also have preferentially reached women with particular educational, professional or sociocultural characteristics. No Aboriginal and Torres Strait Islander women participated in the study, representing an important equity gap that may limit the applicability of these findings to Aboriginal and Torres Strait Islander communities and should be considered when developing and implementing maternal immunisation strategies. Furthermore, the study explored a hypothetical vaccine; therefore, participants’ stated preferences may not translate directly into vaccination behaviour following vaccine introduction. Social desirability may also have influenced responses regarding willingness to vaccinate.

## 5. Conclusions

Pregnant women identified infant protection, vaccine safety, trusted healthcare advice and convenient access as important considerations for future maternal GBS vaccination. These findings highlight the need for implementation planning that combines clear evidence-based communication with accessible, affordable and integrated vaccination pathways within Australian antenatal care.

## Figures and Tables

**Table 1:** Demographic details of participants.

Participant Code	Age	State	Ethnicity	Level of Education	Employment	Gravidity	Gestational Age
P1 (interview 13)	31	SA (Metro)	European	Undergraduate degree	Health sector	First pregnancy	31 weeks
P2 (interview 12)	33	SA (Metro)	European	Undergraduate degree	Health sector	Second pregnancy	28 weeks
P3 (FGD 11)	35	SA (Metro)	European	Postgraduate degree	Health sector	First pregnancy	26 weeks
P4 (FGD 11)	32	SA (Metro)	European	Undergraduate degree	Health sector	Second pregnancy	38 weeks
P5 (FGD 11)	33	SA (Metro)	European	Undergraduate degree	Health sector	Second pregnancy	21 weeks
P6 (FGD 10)	28	SA (Metro)	European	Postgraduate degree	Health sector	First pregnancy	22 weeks
P7 (FGD 10)	31	SA (Metro)	European	Undergraduate degree	Health sector	Second pregnancy	31 weeks
P8 (Interview 9)	33	SA (Metro)	European	Undergraduate degree	Public Administration	Second pregnancy	40 weeks
P9 (Interview 8)	31	SA (Metro)	Middle -Eastern	Postgraduate degree	Health sector	First pregnancy	36 weeks
P10 (Interview 7)	31	SA (Metro)	East Asian	Undergraduate degree	Health sector	First pregnancy	14 weeks
P11 (FGD 6)	34	WA (Metro)	European	Postgraduate degree	Education	Third pregnancy	20 weeks
P12 (FGD 6)	31	SA (Regional)	European	Undergraduate degree	Mining	First pregnancy	33 weeks
P13 (FGD 6)	35	SA (Regional)	European	Year 12	Insurance	Second pregnancy	28 weeks
P14 (FGD 5)	24	VIC (Regional)	African	Year 12	Education	First pregnancy	18 weeks
P15 (FGD 5)	43	TAS (Regional)	European	Postgraduate degree	Education	Second pregnancy	32 weeks
P16 (FGD 5)	32	QLD (Metro)	European	Postgraduate degree	Health sector	Second pregnancy	27 weeks
P17 (FGD 4)	39	SA (Metro)	European	Postgraduate degree	Health sector	Third pregnancy	12 weeks
P18 (FGD 4)	37	SA (Metro)	European	Undergraduate degree	Health sector	Third pregnancy	14 weeks
P19 (FGD 4)	33	NSW (Metro)	South Asian	Postgraduate degree	Health sector	Second pregnancy	9 weeks
P20 (FGD 4)	37	QLD (Regional)	African	Year 12	Not employed	Second pregnancy	12 weeks
P21 (Interview 3)	36	NSW (Metro)	European	Postgraduate degree	Education	Third pregnancy	35 weeks
P22 (FGD 2)	37	NSW (Metro)	European	Undergraduate degree	Legal	Second pregnancy	27 weeks
P23 (FGD 2)	30	NSW (Regional)	East Asian	Postgraduate degree	Health sector	First pregnancy	32 weeks
P24 (FGD 1)	31	ACT (Metro)	East Asian	Postgraduate degree	Education	Second pregnancy	18 weeks
P25 (FGD 1)	33	VIC (Remote)	East Asian	Undergraduate degree	Retail	First pregnancy	35 weeks
P26 (FGD 1)	33	SA (Metro)	European	Postgraduate degree	Education	Second pregnancy	20 weeks
P27 (FGD 1)	37	SA (Metro)	European	Postgraduate degree	Health sector	First pregnancy	18 weeks
P28 (FGD 1)	19	WA (Regional)	European	Year 12	Homemaker	First pregnancy	34 weeks
P29 (FGD 14)	30	WA (Regional)	East Asian	Undergraduate degree	Legal	Second pregnancy	37 weeks
P30 (FGD 14)	33	VIC (Metro)	East Asian	Postgraduate degree	Health sector	Second pregnancy	22 weeks
P31 (FGD 14)	38	NSW (Metro)	South Asian	Postgraduate degree	Information Technology	First pregnancy	33 weeks

Footnote: P, participant; FGD, Focus Group Discussion session.

## Data Availability

De-identified data can be made available to others upon approval by the Women’s and Children’s Hospital Human Research Ethics Committee.

## Supplementary Materials

The following supporting information can be downloaded at https://www.mdpi.com/article/10.3390/vaccines14090807/s1, File S1: Semi-Structured Interview Guide.

## Abbreviations

The following abbreviations are used in this manuscript:

GBS	Group B *Streptococcus*
FGD	Focus Group Discussion
