# Supplementary material for: Preparing for Future Group B Streptococcus Vaccines: Perspectives of Pregnant Women in Australia
Source: Vaccines (Basel). 2026 Sep 14;14(9):807. doi: 10.3390/vaccines14090807 (PMC13611924; doi:10.3390/vaccines14090807)
Supplement: Supplementary file 1 [file vaccines-14-00807-s001.zip › vaccines-4516072-supplementary.pdf]

**File S1-Semi-Structured Interview Guide**

**Introduction, purpose of focus group discussions, confidentiality**

Thank you for agreeing to participate in this focus group discussion on Group B streptococcus infection (commonly called GBS) and future GBS vaccines for pregnant women. Please let me know if you want any questions clarified or if you wish to stop the discussion at any stage. I would also like to let you know that your identity will be known to other focus group participants and the researcher(s) cannot guarantee that others in the group will respect the confidentiality of the group. As a researcher, I ask that you keep all comments made during the focus group confidential and not discuss what happened during the focus group outside the meeting.

We would like to record this interview so that we can be sure we have an accurate record of this conversation. The recording will be transcribed and we will use the data for research. However, you will not be personally identified in the transcript documents, this is to protect your privacy. We will email you a copy of the transcript of this discussion for you to read before we use it for research. Are you happy for us to record this discussion?. This interview will not take more than one hour.

**General introduction**

I'd like each of you to tell a little bit about yourself (state, employment in health sector or not, first pregnancy or not)

**Questions/prompts for the focus group discussion**

**Let's first talk about GBS infection**

1. Have you heard about GBS?
2. what do you know about GBS in pregnancy?

3. During the current pregnancy, did your doctor or midwife tell you about GBS infection?
4. What information would you like to receive during pregnancy about GBS infection?
5. Do you know about the method of screening/testing for GBS in pregnancy?
6. If you test positive for GBS, do you know how it will be managed?
7. What are your thoughts about antibiotics in pregnancy for GBS ?
8. What are your thoughts about antibiotics for newborn babies to treat GBS?

**Future GBS vaccines**

**There are few GBS vaccine that are currently undergoing trials and we expect a vaccine for GBS to be available in the near future. Such a vaccine will be given to pregnant women to prevent their newborn babies from getting GBS infections.**

9. If a GBS vaccine is available for pregnant women, what information would you like to receive before making a decision about getting the vaccine?
10. What would you consider as facilitators for GBS vaccination in pregnancy?
11. What would you consider as barriers for GBS vaccination in pregnancy?
12. Is there anything else that anyone would like to discuss about future GBS vaccines for pregnant women?
